# Tualang Honey Protects the Rat Midbrain and Lung against Repeated Paraquat Exposure

**DOI:** 10.1155/2017/4605782

**Published:** 2017-01-03

**Authors:** Suk Peng Tang, Sirajudeen Kuttulebbai Nainamohamed Salam, Hasnan Jaafar, Siew Hua Gan, Mustapha Muzaimi, Siti Amrah Sulaiman

**Affiliations:** ^1^Department of Pharmacology, School of Medical Sciences, Health Campus, Universiti Sains Malaysia, Kubang Kerian, Kelantan, Malaysia; ^2^Department of Chemical Pathology, School of Medical Sciences, Health Campus, Universiti Sains Malaysia, Kubang Kerian, Kelantan, Malaysia; ^3^Department of Pathology, School of Medical Sciences, Health Campus, Universiti Sains Malaysia, Kubang Kerian, Kelantan, Malaysia; ^4^Human Genome Centre, School of Medical Sciences, Health Campus, Universiti Sains Malaysia, Kubang Kerian, Kelantan, Malaysia; ^5^Department of Neurosciences, School of Medical Sciences, Health Campus, Universiti Sains Malaysia, Kubang Kerian, Kelantan, Malaysia

## Abstract

Paraquat (PQ) is a dopaminergic neurotoxin and a well-known pneumotoxicant that exerts its toxic effect via oxidative stress-mediated cellular injuries. This study investigated the protective effects of Tualang honey against PQ-induced toxicity in the midbrain and lungs of rats. The rats were orally treated with distilled water (2 mL/kg/day), Tualang honey (1.0 g/kg/day), or ubiquinol (0.2 g/kg/day) throughout the experimental period. Two weeks after the respective treatments, the rats were injected intraperitoneally with saline (1 mL/kg/week) or PQ (10 mg/kg/week) once per week for four consecutive weeks. After four weekly exposures to PQ, the glutathione peroxidase activity and the number of tyrosine-hydroxylase immunopositive neurons in the midbrain were significantly decreased in animals from group PQ (*p* < 0.05). The lungs of animals from group PQ showed significantly decreased activity of superoxide dismutase and glutathione-S-transferase. Treatment with Tualang honey ameliorated the toxic effects observed in the midbrain and lungs. The beneficial effects of Tualang honey were comparable to those of ubiquinol, which was used as a positive control. These findings suggest that treatment with Tualang honey may protect against PQ-induced toxicity in the rat midbrain and lung.

## 1. Introduction

Paraquat (PQ), or 1,1′-dimethyl-4,4′-bipyridinium, is a nonselective contact herbicide commonly used to control broadleaf and grassy weeds. Currently, it is among the most frequently used weed killers in approximately 100 countries. Since its first introduction in the early 1960s, problems resulting from PQ use, for example, suicidal intent, accidental poisoning, or occupational exposure, have frequently been reported [1].

PQ is a strong electron acceptor and readily undergoes a single-electron reduction to form the monocation radical (PQ^+*∙*^). This radical is rapidly reoxidized by molecular oxygen, regenerating the dicationic form (PQ^2+^) and generating a superoxide radical (O_2_^∙^). The process of PQ redox cycling and the initiation of the cascade reaction by O_2_^∙−^ leads to the formation of various reactive oxygen species (ROS) [2, 3], which readily attack key cellular structures and molecules (lipids, carbohydrates, proteins, and nucleotides), leading to deleterious cellular effects underlying various diseases [4].

The effect of chronic exposure to PQ, particularly among agricultural workers and PQ formulation workers, has gained considerable attention due to the wide use of PQ around the world. Unlike the major pneumotoxic effects of acute poisoning [2, 5], repeated low-dose exposure to PQ is believed to be neurotoxic and may be involved in the pathogenesis of Parkinson's disease [6, 7]. There is growing evidence from epidemiological studies indicating the possible involvement of environmental toxins, such as pesticides, as an important etiological factor for Parkinson's disease. Studies conducted by Liou et al. [8] and Hertzman et al. [9] showed that exposure to PQ was associated with increased incidence of Parkinson's disease.

PQ first received research interest as a potential neurotoxin due to its structural similarity to 1-methyl-4-phenylpyridinium (MPP^+^), a known dopaminergic neurotoxin that induces Parkinson-like symptoms in both humans and experimental animals [10, 11]. The brain naturally contains relatively low levels of antioxidants and high amounts of polyunsaturated fatty acids, making it more susceptible to oxidative injuries from redox imbalance [12]. Of the neuronal cell types in the brain, dopaminergic neurons in the nigrostriatal system are selectively vulnerable to oxidative injury because dopamine metabolism itself generates high levels of ROS [7, 13]. A recent report by Pacelli et al. [14] demonstrated that dopaminergic neurons of the substantia nigra pars compacta (SNpc) have a more complex axonal arborization and denser populations of axonal mitochondria and therefore have higher basal energy demands, consequently placing these neurons at higher risk in Parkinson's disease. PQ-induced oxidative stress has been demonstrated in the substantia nigra (SN) in Parkinson's disease brain [15]. In experimental models, rats or mice exposed to PQ demonstrate a selective degeneration of nigrostriatal dopaminergic neurons, a neuropathological hallmark of Parkinson's disease [13, 16].

Because the suggested primary mechanism for PQ toxicity, either acute or chronic, involves the production of ROS, it is plausible that an antidote against PQ poisoning should be a substance that can confer strong antioxidant properties. Honey is known to be rich in both enzymatic and nonenzymatic antioxidants, including both aqueous and lipophilic antioxidants, and the interactions among these antioxidants suggest that honey may be an ideal natural antioxidant that can act at multiple subcellular locations in the case of PQ poisoning [17, 18].

Tualang honey is a wild honey harvested from Tualang trees found in the Malaysian rain forest. Various studies have been conducted to evaluate its possible medicinal uses, including anticancer properties of Tualang honey in cell culture and animal models [19, 20], protective effects against damage induced by cigarette smoke on the rat male reproductive system [21], an animal model of menopause [22], an animal model of diabetes [23, 24], and its wound healing and antimicrobial properties [25–27].

The neuroprotective effects of Tualang honey have previously been reported in chronic cerebral hypoperfusion-induced neurodegeneration in the rat hippocampus [28]. In addition, in stressed ovariectomized rats, supplementation of Tualang honey improved the morphology of the hippocampus and medial prefrontal cortex, the performance of memory tasks, and the cholinergic system [29, 30]. However, to our knowledge, there is no study reporting on the potential beneficial effects of Tualang honey against PQ-induced neurotoxicity. In addition, the effects of Tualang honey against PQ-induced pneumotoxicity have also been evaluated because PQ is a well-known pneumotoxicant. Therefore, the objective of this study is to investigate the possible neuroprotective and pneumoprotective effects of Tualang honey in rats exposed to four weekly injections of PQ.

## 2. Materials and Methods

### 2.1. Ethics Statement

The ethical approval for this study was obtained from the Animal Ethics Committee, Universiti Sains Malaysia (USM) [Approval No.: USM/Animal Ethics Approval/2012/(75)(398)] in accordance with the Institutional Guidelines for the Care and Use of Animals for Scientific Purposes. Eight-week-old male Sprague-Dawley rats were purchased from the Animal Research and Service Centre, Health Campus, USM, Kubang Kerian, Kelantan, Malaysia. All animals were individually housed in a well-ventilated animal room maintained with a 12-hour/12-hour light/dark cycle and a temperature of 25 ± 2°C. The animals were allowed access to food pellets and water ad libitum unless otherwise stated. All rats were acclimatized to the animal room conditions for at least one week before the experiments started.

### 2.2. Tualang Honey

The Tualang honey (AgroMas®) used in this study was supplied by the Federal Agricultural Marketing Authority (FAMA), Kedah, Malaysia. The honey samples were filtered, concentrated to 20% (w/v) water content at 40°C, and sterilized by gamma irradiation at 25 kGy [Sterilgamma (M) Sdn. Bhd., Selangor, Malaysia]. The same batch of honey was used throughout the analysis.

### 2.3. Experimental Design

**Figure 1:**
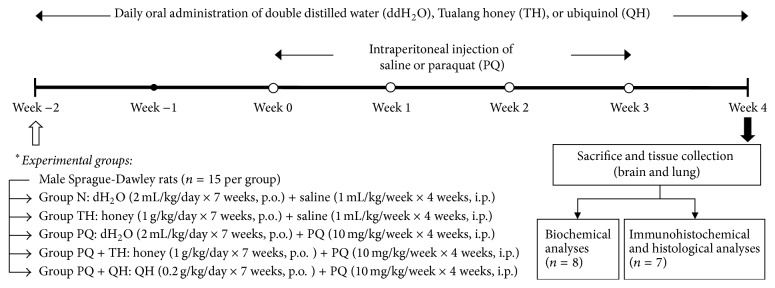
Experimental procedure. A total of 75 rats were randomly divided into five groups of 15 animals each and were orally treated with distilled water (dH_2_O, 2 mL/kg/day), Tualang honey (TH, 1 g/kg/day), or ubiquinol (QH, 0.2 g/kg/day) throughout the experimental period. Two weeks after these treatments, the rats were injected with saline (1 mL/kg/week) or PQ (PQ, 10 mg/kg/week) once a week (open circles) for four consecutive weeks. A week after the last injection, the rats were sacrificed for biochemical (*n* = 8) or immunohistochemical (IHC, *n* = 7) analyses.

A total of 75 rats were randomly divided into five groups of 15 animals each: control (N), honey (TH), paraquat (PQ), paraquat + honey (PQ + TH), and paraquat + ubiquinol (PQ + QH). Throughout the experiment, groups N and PQ were treated with distilled water (2.0 mL/kg/day, p.o.), groups TH and PQ + TH were treated with Tualang honey (AgroMas, FAMA, 1.0 g/kg/day, p.o.), and group PQ + QH was treated with ubiquinol (0.2 g/kg/day, p.o.) once per day (Figure 1). The dose of Tualang honey was chosen based on previous studies that showed protective effects against other oxidative stress-related diseases [21, 31–33], while the selected ubiquinol dose was based on the studies by Cleren et al. [34] and Matthews et al. [35]. Two weeks after the respective treatments, rats were administered intraperitoneal injections of vehicle (0.9% NaCl, 1.0 mL/kg; Group N and TH) or PQ (Sigma-Aldrich, 10 mg/kg; Group PQ, PQ + TH and PQ + QH) once per week for four consecutive weeks. One week after the final injection of saline or PQ, the rats were fasted overnight and sacrificed for biochemical (*n* = 8 per group) or immunohistochemical (*n* = 7 per group) analyses. Body weight changes, renal profile, and liver profile were included as general health assessments.

### 2.4. Biochemical Analysis

#### 2.4.1. Sample Collection

The rats (*n* = 8 per group) were fasted overnight and were deeply anaesthetized with intramuscular injection of ketamine (90 mg/kg) plus xylazine (5 mg/kg). Blood samples (5 mL) were collected via the portal vein into plain tubes and were allowed to clot at 4°C before being centrifuged at 3000 ×g for 15 min. The serum yielded was aliquoted and temporarily stored at 4°C before being outsourced to a private laboratory (BP Healthcare, Malaysia) for tests of renal and liver function. Immediately after blood collection, brain and lung samples were rapidly dissected out, rinsed with ice-cold phosphate-buffered saline (PBS) (1x, pH 7.4) to remove any residual blood, and stored at −80°C for subsequent biochemical analyses.

#### 2.4.2. Preparation of Tissue Homogenate

The midbrain region was identified and dissected according to the method described by Heffner et al. [36]. The harvested midbrain region and the right lung were homogenized in ice-cold potassium phosphate buffer (0.1 M, pH 7.4) to produce a 10% (w/v) homogenate using a motor-driven Teflon-glass homogenizer. The samples were then centrifuged at 10,000 ×g for 10 min at 4°C. Aliquots of the resulting supernatant were used for the measurement of total protein; the activities of superoxide dismutase (SOD), catalase (CAT), glutathione peroxidase (GPx), glutathione reductase (GR), and glutathione-S-transferase (GST); and the total levels of glutathione (GSH) and malondialdehyde (MDA), using commercially available assay kits. The assay kits for quantifying total protein, SOD, GPx, GR, GST, total GSH, and MDA were purchased from Cayman Chemical Company (USA). The assay kit for CAT was purchased from BioAssay Systems (USA). Additionally, the midbrain tyrosine hydroxylase (TyrH) concentration was measured using an ELISA kit from Cusabio Biotech, China.

### 2.5. Immunohistochemical Analysis of Tyrosine Hydroxylase

#### 2.5.1. Sample Collection

The rats (*n* = 7 per group) were fasted overnight, deeply anesthetized with ketamine/xylazine mixture, and perfused transcardially with prechilled PBS buffer, followed by 4% paraformaldehyde prepared in 0.1 M phosphate buffer (pH 7.4) [37]. The brains were removed from the cranium and postfixed in the same fixative for another 48–72 hours at 4°C before being processed for tyrosine hydroxylase (TyrH) immunohistochemistry.

#### 2.5.2. Tyrosine Hydroxylase Immunohistochemistry

Midbrain dopaminergic neurons were stained with TyrH antibody for evaluation of midbrain damage based on the number of TyrH-positive neurons in the SNpc region. After the fixation period, the striatum and midbrain were identified and dissected according to the rat brain atlas [38]. The coronal sections containing the striatum (anterior-posterior [A-P] = +1.5 to −1.0 mm from bregma) and substantia nigra (AP = −4.8 to −6.5 mm from bregma) were collected for immunohistochemical (IHC) staining of TyrH. The sectioned tissues were processed using an automated tissue processor before being embedded in paraffin blocks. Tissue sections (3 *μ*m thick) were prepared using a rotary microtome and were then mounted on poly-L-lysine-coated slides. Prior to IHC staining of TyrH, the slides were heated overnight in an oven at 60°C to increase tissue adherence. A tissue section from the striatum (as a positive control) and a tissue section from the liver (as a negative control) were included in each batch of staining.

Briefly, the tissue sections were deparaffinized with xylene, rehydrated in decreasing concentrations of ethanol, and washed with 1x Tris buffered saline (TBS) wash buffer (pH 7.6; Dako, Denmark) and double-distilled water (ddH_2_O). The rehydrated tissue sections were microwaved with citrate buffer (pH 6; Dako, Denmark) for antigen retrieval, blocked with peroxidase-blocking solution (REAL™, Dako, Denmark), and incubated overnight with TyrH antibody raised in rabbit (AB152, Millipore, 1 : 10,000 dilution) at 4°C. The tissues were then rinsed with 1x TBS wash buffer, incubated with horseradish peroxidase- (HRP-) conjugated secondary antibody, and exposed to diaminobenzidine (DAB) as a substrate (REAL EnVision Detection Systems, K5007, Dako, Denmark). The HRP reacts with the DAB substrate to produce a brown product. The slides were then counter-stained with hematoxylin (Sigma-Aldrich, USA), subsequently dehydrated in increasing concentrations of ethanol, cleared with xylene, and sealed with coverslips. The slides were examined under a light microscope (Olympus BX41, Japan). The number of TyrH-positive neurons in the SNpc region was determined in three sections from each rat. The SNpc was delineated with reference to the rat brain atlas [38]. The TyrH-positive neurons were identified by dense cytoplasmic immunolabeling.

### 2.6. Statistical Analysis

Statistical analysis was performed using IBM SPSS software version 22.0 (Armonk, NY, USA). Data that were normally distributed are presented as the mean (standard deviation). The differences among the means of the groups were analyzed using one-way analysis of variance (ANOVA). If the ANOVA showed a significant difference, Tukey's post hoc test was performed to determine the pairwise differences of the means. Data that were not normally distributed are expressed as the median (interquartile range [IQR]). The differences among the medians of the groups were analyzed using the Kruskal-Wallis test, and if significant, the Mann-Whitney *U*-test was performed for pairwise comparisons of the medians. Differences were considered statistically significant when *p* < 0.05.

## 3. Results

### 3.1. Changes in Body Weight

**Figure 2:**
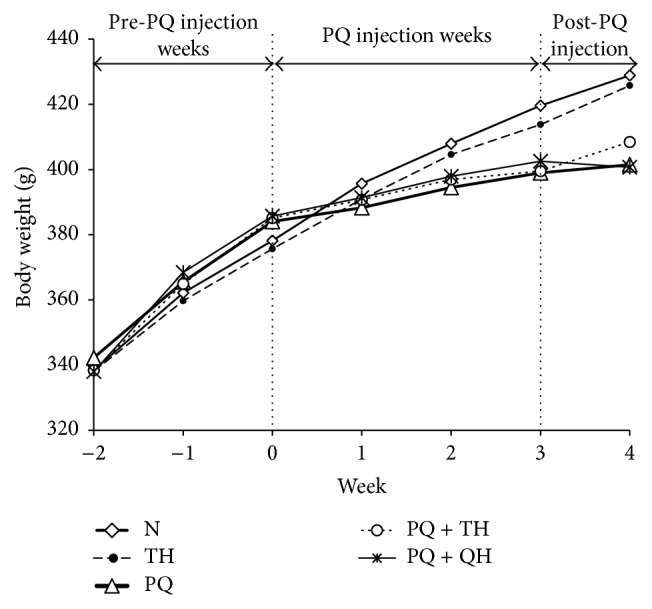
Mean body weight (g) of rats throughout the experimental period (*n* = 15 per group). Overall, the body weight of rats from all experimental groups showed an increasing trend throughout the experimental period.

Generally, the body weight of rats from all experimental groups progressively increased (Figure 2). However, the body weight gain in all PQ-treated groups (PQ, PQ + TH, and PQ + QH) was significantly lower than in the vehicle control groups (N and TH) (*p* = 0.003 at week 1 and *p* < 0.001 at weeks 2, 3, and 4 when compared to week 0; Table 1). Treatment with Tualang honey or ubiquinol did not significantly affect the body weight of PQ-intoxicated rats.

### 3.2. Serum Biochemical Parameters

#### 3.2.1. Renal Profile

No significant differences in the serum levels of sodium, potassium, chloride, and uric acid were observed among the experimental groups. The rats that received honey (groups TH and PQ + TH) and ubiquinol (groups PQ + QH) showed a significant decrease in the serum urea level (*p* < 0.05 for group TH, *p* < 0.005 for groups PQ + TH and PQ + QH) compared to that in group N. The serum urea level in group PQ + TH was also significantly lower than that in group PQ (*p* < 0.05). In addition, the serum creatinine level in group PQ + TH was significantly lower than in groups N (*p* < 0.01) and TH (*p* < 0.05) (Table 2).

#### 3.2.2. Liver Profile

No significant differences were observed for serum levels of total protein or for aspartate transaminase (AST) or alkaline phosphatase (ALP) activity in any of the experimental groups. However, serum alanine transaminase (ALT) activity was significantly higher in group PQ than in group TH (*p* < 0.05). Treatment with honey (group PQ + TH) significantly reduced ALT activity in PQ-intoxicated rats (*p* < 0.005) (Table 2).

### 3.3. The Effects of PQ and Tualang Honey on the Rat Midbrain

#### 3.3.1. Assessment of Oxidative Stress Parameters

The present study showed that there were no significant differences in the enzymatic activities of SOD, CAT, GR, and GST in the midbrain region among all experimental groups. However, the midbrain GPx activity was significantly lower in groups PQ and PQ + QH (*p* < 0.05; Table 3). Measurement of the total GSH level also showed no significant differences among the investigated groups. The level of MDA, a marker for lipid peroxidation, was higher in group PQ than in all other experimental groups, although this difference did not reach statistical significance (*p* = 0.074).

#### 3.3.2. Assessment of Damage to Midbrain Dopaminergic Neurons

**Figure 3:**
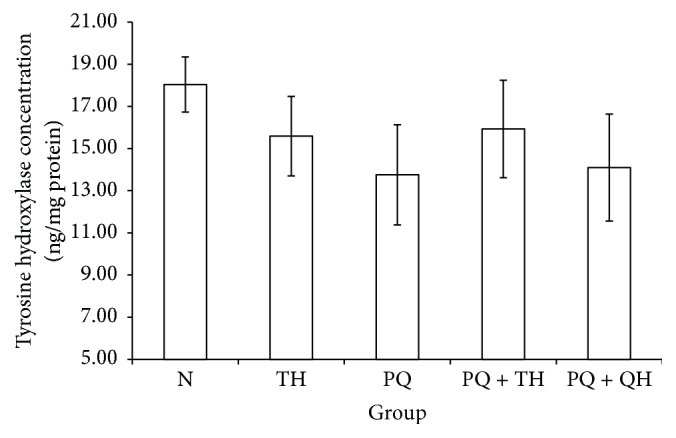
Midbrain concentrations of TyrH for all experimental groups. Data are presented as the mean ± SEM, *n* = 8 per group. No significant differences (*p* = 0.637) were observed among all the experimental groups.

**Figure 4:**
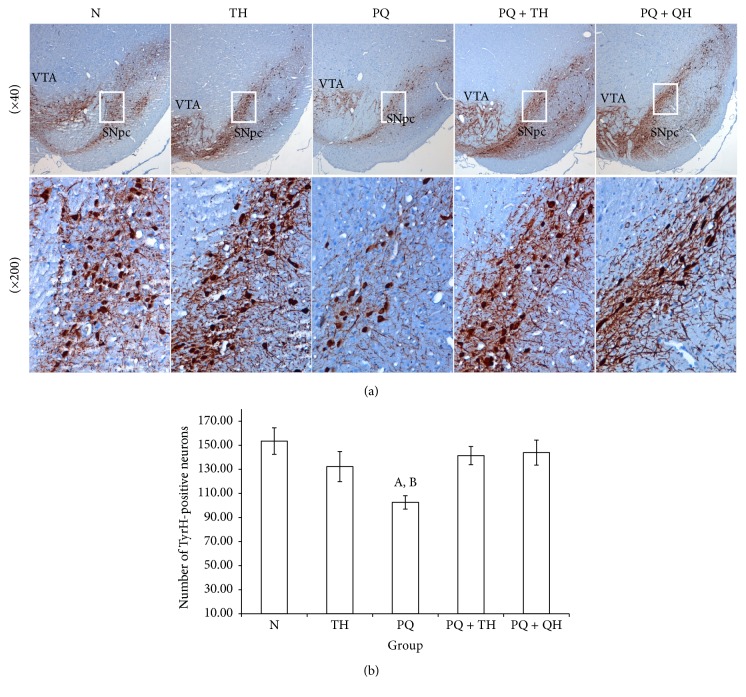
TyrH immunohistochemical staining for evaluating damage to dopaminergic neurons. (a) Representative photomicrographs showing TyrH-immunopositive neurons in coronal sections showing the midbrain. The higher magnification (×200) for the boxed region shows the reduction in TyrH-positive neurons. (b) The numbers of TyrH-positive neurons in the SNpc regions were counted. Data are presented as the mean ± SEM, *n* = 7 per group. The number of TyrH-positive neurons was significantly lower in PQ-intoxicated rats than in group N (^A^*p* = 0.007). Compared with group PQ, the number of TyrH-positive neurons increased in group PQ + TH, although this difference is not statistically significant (*p* = 0.059). Group PQ + QH showed a significant increase in the number of TyrH-positive neurons compared to group PQ (^B^*p* = 0.039).

Damage to midbrain dopaminergic neurons was assessed via measurement of the midbrain TyrH concentration and immunohistochemical assessment of TyrH-positive neurons in the SNpc. None of the experimental groups showed a significant change in the mean TyrH level (Figure 3). Microscopic examination of TyrH-positive neurons in the SNpc (Figure 4(a)) showed a decreased number of TyrH-positive neurons in group PQ. Neuronal loss was also observed in group PQ based on a decrease in TyrH-positive fibers. The number of TyrH-positive neurons counted in the three midbrain sections containing the SNpc was significantly lower in group PQ than in group N (*p* = 0.007). However, treatment with QH significantly increased the number of TyrH-positive neurons (*p* = 0.039). The number of TyrH-positive neurons also increased in group PQ + TH, although this difference did not reach statistical significance (*p* = 0.059) (Figure 4(b)).

### 3.4. The Effects of PQ and Tualang Honey on Rat Lung

#### 3.4.1. Assessment of Oxidative Stress Parameters

PQ-intoxicated rats (group PQ) showed significantly lower total SOD activity than the control (*p* < 0.005) and honey (*p* < 0.05) groups. The PQ-intoxicated rats that received honey (*p* < 0.05) or ubiquinol treatments (*p* < 0.05) showed significantly greater SOD activity, at levels comparable to that of the vehicle control group (*p* > 0.05). No significant differences were observed in the mean GST activity among the investigated groups, except for group PQ. GST activity was significantly lower in PQ-treated rats than in group N (*p* < 0.005). In addition, the total GSH level in the lungs was also lower in group PQ than in groups N and TH, although this difference is not statistically significant (*p* > 0.05). However, the total GSH level in the lungs was significantly higher in group PQ + QH than in group PQ (*p* < 0.05). No significant differences among the experimental groups were observed for the other parameters (Table 4).

## 4. Discussion

Our study is the first to show that Tualang honey may provide some protection to the rat midbrain and lung following repeated exposure to PQ. In the present study, all groups of rats treated with PQ (PQ, PQ + TH, and PQ + QH) showed significantly reduced rates of body weight gain compared to that in the vehicle control groups (N and TH). The significantly smaller gains in body weight were observed as early as one week following the first PQ administration. The overall slower body weight gain may be attributable to the lower food intake, particularly in the first two to three days following PQ administration (data not shown). PQ-induced conditioned taste aversion and weight loss have previously been shown to be mediated by the area postrema of the hypothalamus [39, 40]. Studies by Chanyachukul et al. [41] suggest a PQ-induced significant reduction of noradrenaline content in the hypothalamus that may contribute to the taste aversion and weight loss in rats. However, no significant differences in the total food intake among the experimental groups were observed in this study.

Abnormal renal and liver functions have often been observed in animal models of PQ poisoning [42, 43]. In the present study, the serum biochemical parameters did not differ significantly between the control (N) and paraquat-treated (PQ) group. However, Tualang honey treatment (group TH) produced a significant reduction in serum urea content when compared to that of group N, as well as in group PQ + TH when compared to that of group PQ. The serum creatinine level was also markedly lower in group PQ + TH than in groups N and TH. In addition, the two groups receiving honey (TH and PQ + TH) showed significantly lower ALT activity than group PQ. Similarly, lower AST activity was observed in groups TH and PQ + TH than in group PQ. Similar to our findings, Al-Waili [44] previously reported that a daily consumption of honey for two weeks increased blood levels of antioxidants and could even decrease the levels of the liver enzymes AST and ALT in normal individuals. Nonsignificant decrease in the levels of blood urea nitrogen and serum creatinine has also previously been reported among normal subjects receiving honey [45], indicating that honey may confer some protective effects on the affected organs due to its high antioxidant content [46]. In animal models, honey administration reduced blood urea nitrogen, serum creatinine, AST, and ALT in healthy sheep and showed hepatoprotective effects against carbon tetrachloride-induced liver injury [47]. A recent study by Tanvir et al. [48] showed that honey supplementation can ameliorate chlorpyrifos-induced renal and liver toxicities in rats, which was mainly attributed to the antioxidant properties of the honey. The hepatoprotective effects of Tualang honey were previously observed in streptozotocin- (STZ-) induced diabetic rats [33]. However, because the present study did not include the serum levels of antioxidants and oxidative stress markers, further investigations are needed to understand the exact mechanism of the protective function observed here.

Following four weekly systemic administrations of PQ to rats, oxidative injuries to the midbrain were demonstrated by significantly lower GPx activity than in the control group. In addition, group PQ also showed lower activities of SOD and CAT, a lower total GSH level, and a higher level of MDA compared to the control group, although these differences were not statistically significant. No significant differences in the midbrain TyrH concentration were observed among the experimental groups. However, immunohistochemical assessment of the SNpc showed a significant reduction in the number of TyrH-positive neurons in group PQ, indicating damage to dopaminergic neurons. Pretreatment with Tualang honey or ubiquinol two weeks prior to injection of PQ ameliorated the toxic effects of PQ, suggesting that Tualang honey or ubiquinol protects dopaminergic neurons against oxidative stress-related damage, possibly via an antioxidant defense mechanism.

As mentioned above, PQ readily undergoes cellular redox cycling to induce the generation of free radicals. In addition, an increased rate of dopamine oxidation was previously reported in the SN of mice following repeated PQ injections (i.p., twice weekly for three consecutive weeks) [49], which could also serve as an additional source for ROS production. Excessive generation of free radicals is one plausible explanation for the decrease in SOD and GPx activities in group PQ. The depletion of these antioxidants in group PQ may render the midbrain region more vulnerable to oxidative damage [50, 51]. Similarly, a study conducted by Mehdi and Qamar [52] showed that brain SOD activity in* D. melanogaster* was decreased by PQ in a dose-dependent manner and that the decrease in SOD activity was strongly associated with increased oxidative stress and neuronal DNA damage.

The catalytic actions of several enzymes including SOD and monoamine oxidase (an enzyme involved in dopamine metabolism) generate hydrogen peroxide (H_2_O_2_), a relatively stable and diffusible ROS [53]. CAT and GPx are two major antioxidant enzymes that work together to detoxify H_2_O_2_ to water and oxygen. In this study, although the differences among the experimental groups were not significant, the midbrain CAT activity was lower in group PQ and was higher in the two groups that received Tualang honey (groups TH and PQ + TH) than in the control group (N). Increased CAT activity following honey supplementation has previously been reported in animal [54] and human disease models [55, 56].

In comparison to the relatively unchanged midbrain CAT activity observed, significant decreases in GPx activity were observed in groups PQ and PQ + QH. The catalytic activity of GPx requires coupled reactions with GSH redox cycling during the decomposition of peroxides [57]. It has been suggested that, at low concentrations of peroxides and high GSH level, GPx decomposes H_2_O_2_ faster than CAT [58]. Unlike GPx, CAT catalyzes the decomposition of H_2_O_2_ without requiring a hydrogen donor; therefore, it may play a more important role during clearance of high concentrations of H_2_O_2_, thus ensuring that the decomposition of peroxides still occurs when GSH is depleted [58, 59]. In addition, GPx and GSH are found mainly in the cytosol and mitochondria and may therefore respond more quickly to extracellular H_2_O_2_ in comparison to CAT, which is more restricted to peroxisomes [60, 61]. Taken together, these phenomena may explain the significant reduction in GPx but not CAT in the present study, given that GPx may respond faster than CAT, particularly during the early stages of the disease. A recent study conducted by Gobbo et al. [62] showed similar findings, with activation of GPx occurring early during the response to diabetes, whereas activation of CAT tended to occur at later stages of untreated diabetes. Further research is needed to understand the effects of PQ on GPx and CAT activities in the midbrain.

GSH is the major low molecular weight thiol in mammals and is one of the most important antioxidants in the brain. In addition to serving as a substrate for GPx activity, GSH also plays important roles in cellular detoxification processes through conjugation with various endogenous and xenobiotic compounds; these functions are mediated by GST [63]. In other words, GST play an important role in protecting cells from injury by toxic chemicals and oxidative stress products. Indeed, increased GST-pi isoenzyme expression following systemic administration of MPTP was previously shown, whereby GST-pi expression was suggested to be part of the protective mechanism against MPTP-induced dopaminergic neurodegeneration [64]. In this study, GST activity was higher in group PQ than in the control group, although this difference was not significant. Along with the slight decrease in GSH observed in group PQ, this suggests that GST may be involved in the detoxification of PQ via the conjugation of PQ to GSH. GSH depletion without any significant changes in GST activity has previously been reported in the SN in Parkinson's disease [65]. Higher GR activity, although not statistically significant, was also observed in group PQ, suggesting that the catalytic activity of GR increased to restore the marginally depleted GSH in group PQ.

Despite the significant decreases in GPx activity observed in groups PQ and PQ + QH, a significant loss of TyrH-positive neurons was only observed in group PQ. In addition, group PQ showed the lowest GSH level among all of the experimental groups, and treatment with honey or ubiquinol ameliorated the decrease in the GSH level. Taken together, these findings may explain the importance of GSH in protecting SNpc dopaminergic neurons [66] from PQ toxicity.

Decreased numbers of TyrH-positive neurons in the SNpc have also previously been reported in an animal model of PQ-induced neurotoxicity [16, 49, 67, 68]. It was suggested that the compensatory mechanisms involving dopaminergic and noradrenergic transmission as well as nondopaminergic pathways were activated three days after PQ withdrawal [16]. For instance, when dopamine levels decrease due to the loss of dopaminergic neurons, the surviving neurons may rapidly upregulate TyrH to compensate for the loss [69]. The activation of compensatory mechanisms may likely explain the significant reduction in the number of TyrH-positive neurons but not in the midbrain TyrH level in animals that received PQ. In addition, because TyrH concentrations were measured using the whole midbrain and were not restricted to the SNpc as in the immunohistochemical analysis, these changes may not represent the actual changes in the SNpc.

Due to marked respiratory distress observed in our preliminary study (data not shown), the effects of four weekly PQ injections on the lungs were also evaluated in this study. Most of the published research has focused on the effects of systemic administration of PQ on dopaminergic neurons [16, 70, 71], and relatively few reports include the effects of PQ on both dopaminergic neurons and on lung toxicity [72–74]. In the present study, the four weekly intraperitoneal injections of PQ caused significant decreases in the activity levels of SOD and GST, along with a lower CAT activity and a lower total GSH content when compared to the control group, thus suggesting that PQ-induced ROS generation leads to depletion of these antioxidant pools. However, pretreatment with Tualang honey or ubiquinol ameliorates the toxic effects of PQ, probably by restoring the depleted antioxidant pools. However, no significant increase in lipid peroxidation was observed via measuring MDA levels. In addition, clinical observation of the rats during the experimental period did not reveal any signs of respiratory distress. A prolonged observation period may be necessary to confirm the development of respiratory failure, a frequent delayed fatal outcome in subacute PQ toxicity, because significant changes in certain oxidative markers were observed.

Tualang honey has previously been reported to be a good source of phenolic compounds [46, 75] and to have strong antioxidant properties [76, 77], suggesting that the protective effects observed occur via antioxidant mechanisms, thus conferring subsequent protection against oxidation-related damage. For instance, pretreatment with kaempferol, one of the flavonoids found in Tualang honey, has been reported to increase the activities of SOD and GPx, reduce the MDA content in the SN, and even prevent TyrH-positive neuronal loss in an MPTP-induced mouse model of Parkinson's disease [78]. However, further investigations are needed to elucidate the exact mechanism by which Tualang honey protects against PQ-induced oxidative stress and related damage in various cell types. A more prolonged experimental period may be needed to elucidate the exact biochemical changes involved, especially at the later stages of the disease. In addition, the present study mainly focuses on the effects of PQ in the region of the midbrain where the cell bodies of the dopaminergic neurons reside. Measurement of striatal dopamine concentrations and motor function assessment may help to determine the functions of dopaminergic terminals.

## 5. Conclusions

In conclusion, our current study model showed that four weekly intraperitoneal injections of PQ elicited early oxidative stress in both the midbrain and the lungs of exposed rats. Pretreatment with Tualang honey or ubiquinol (positive control) ameliorated the toxic effects of PQ observed in both regions. Tualang honey has the potential to ameliorate oxidative stress, as seen in the midbrain and lung of rats after repeated exposure to PQ.

## Figures and Tables

**Table 1:** Body weight changes (percentage relative to week 0, injection week) in all experimental groups.

Week	Body weight changes relative to week 0 (%)					*p* value^*∗*^
	N	TH	PQ	PQ + TH	PQ + QH	
1	3.97 (5.20)	3.94 (2.56)	2.36 (5.51)^a,e^	2.36 (4.40)^a,f^	2.33 (1.71)^b,g^	0.003
2	7.79 (6.50)	7.45 (3.63)	5.51 (7.25)^a,e^	4.94 (6.93)^b,g^	3.79 (2.78)^c,h^	<0.001
3	11.02 (4.86)	10.32 (7.48)	6.96 (7.54)^c,e^	7.19 (12.58)^b,e^	4.21 (4.97)^c,g^	<0.001
4	13.90 (4.00)	13.43 (8.18)	8.12 (10.95)^c,g^	8.50 (13.38)^a,e^	4.75 (7.02)^d,h^	<0.001

Data are expressed as the median (IQR), *n* = 15 per group.

^*∗*^ *p* values were calculated using the Kruskal-Wallis test. A *p* value < 0.05 indicates a statistically significant difference between the investigated groups.

^a^ *p* < 0.05, ^b^*p* < 0.01, ^c^*p* < 0.005, and ^d^*p* < 0.001 compared to group N; ^e^*p* < 0.05, ^f^*p* < 0.01, ^g^*p* < 0.005, and ^h^*p* < 0.001 compared to group TH.

**Table 2:** Serum biochemical parameters in all the experimental groups.

Parameters	Groups					*p* value^*∗*^
	N	TH	PQ	PQ + TH	PQ + QH	
^¶^Sodium (mmol/dL)	139.00 (2.00)	138.00 (4.00)	140.00 (6.00)	140.00 (5.00)	140.00 (2.00)	0.129
^#^Potassium (mmol/dL)	5.11 (0.23)	5.08 (0.37)	5.10 (0.40)	4.80 (0.38)	4.65 (0.50)	0.069
^#^Chloride (mmol/dL)	101.50 (2.67)	101.00 (2.51)	102.00 (1.69)	101.38 (3.38)	103.00 (2.51)	0.595
^¶^Urea (mg/dL)	38.00 (6.00)	29.50 (8.00)^a^	34.50 (6.00)	29.50 (4.00)^c,e^	31.50 (8.00)^c^	0.005
^¶^Creatinine (mg/dL)	0.50 (0.00)	0.50 (0.00)	0.50 (0.10)	0.40 (0.10)^b,d^	0.50 (0.10)	0.039
^¶^Uric acid (mg/dL)	1.20 (0.40)	1.15 (0.70)	1.15 (0.40)	1.00 (0.30)	1.00 (0.30)	0.384
^#^Total protein (g/dL)	5.91 (0.32)	5.83 (0.30)	5.75 (0.42)	5.59 (0.39)	5.56 (0.28)	0.217
^¶^AST (U/L)	77.00 (42.00)	72.00 (17.00)	85.50 (40.00)	72.00 (17.00)	66.50 (21.00)	0.236
^¶^ALT (U/L)	34.00 (15.00)	33.00 (14.00)^e^	40.00 (6.00)	29.50 (5.00)^f^	31.50 (7.00)	0.049
^#^ALP (U/L)	127.00 (34.43)	112.13 (22.66)	118.13 (21.46)	113.38 (22.45)	106.13 (27.38)	0.590

Data are presented as the ^#^mean (SD) or the ^¶^median (IQR), *n* = 8 per group.

^*∗*^ *p* values were calculated using one-way ANOVA or the Kruskal-Wallis test. A *p* value < 0.05 indicates a statistically significant difference between the investigated groups.

^a^ *p* < 0.05, ^b^*p* < 0.01, and ^c^*p* < 0.005 compared to group N; ^d^*p* < 0.05 compared to group TH; ^e^*p* < 0.05, ^f^*p* < 0.005 compared to group PQ.

**Table 3:** Comparison of oxidative stress parameters in the midbrain among all experimental groups.

Parameters	Groups					*p* value^*∗*^
	N	TH	PQ	PQ + TH	PQ + QH	
^#^SOD (U/mg protein)	52.54 (5.57)	51.60 (8.07)	48.17 (5.31)	52.76 (6.43)	52.90 (8.35)	0.619
^#^CAT (U/mg protein)	23.85 (4.30)	27.09 (9.23)	20.64 (5.41)	26.45 (7.48)	21.86 (5.74)	0.248
^#^GPx (U/mg protein)	43.06 (3.52)	40.48 (3.78)	37.50 (4.81)^a^	40.50 (2.77)	37.55 (3.88)^*∗*^	0.031
^¶^GR (U/mg protein)	12.71 (2.85)	13.72 (3.62)	14.72 (1.80)	13.49 (3.91)	13.03 (4.24)	0.530
^#^GST (U/mg protein)	37.05 (4.42)	37.67 (3.91)	38.68 (3.05)	36.13 (4.03)	37.05 (2.92)	0.727
^#^Total GSH (*μ*M/mg protein)	362.05 (64.49)	375.10 (38.48)	340.45 (72.31)	381.39 (37.93)	377.68 (24.45)	0.495
^#^MDA level (*μ*M/mg protein)	0.70 (0.29)	0.69 (0.26)	1.09 (0.67)	0.65 (0.13)	0.58 (0.27)	0.074

Data are presented as the ^#^mean (SD) or the ^¶^median (IQR), *n* = 8 per group.

^*∗*^ *p* values were calculated using one-way ANOVA or the Kruskal-Wallis test. A *p* value < 0.05 indicates a statistically significant difference between the investigated groups.

^a^ *p* < 0.05 compared to group N.

**Table 4:** Comparison of oxidative stress parameters in the lungs among all experimental groups.

Parameters	Groups					*p* value^*∗*^
	N	TH	PQ	PQ + TH	PQ + QH	
^¶^SOD (U/mg protein)	20.75 (6.40)	18.53 (3.03)^b^	17.39 (2.76)^a^	19.25 (3.93)^b^	20.37 (9.53)^b^	0.019
^#^CAT (U/mg protein)	95.35 (12.05)	97.12 (8.86)	79.23 (17.46)	96.71 (19.61)	95.40 (13.83)	0.102
^#^GPx (U/mg protein)	227.95 (29.88)	220.38 (23.96)	205.88 (25.89)	209.59 (29.98)	227.60 (31.93)	0.409
^#^GR (U/mg protein)	19.04 (3.62)	19.09 (2.38)	17.73 (2.76)	19.63 (2.54)	18.31 (2.43)	0.689
^#^GST (U/mg protein)	22.65 (1.33)	20.74 (1.81)	16.73 (5.26)^a^	20.36 (1.90)	18.80 (3.41)	0.008
^#^Total GSH (*μ*M/mg protein)	135.78 (33.60)	112.03 (29.45)	90.43 (35.07)	120.52 (16.77)	143.77 (53.12)^b^	0.043
^#^MDA level (*μ*M/mg protein)	1.42 (0.14)	1.63 (0.32)	1.46 (0.14)	1.39 (0.33)	1.57 (0.27)	0.312

Data are presented as the ^#^mean (SD) or the ^¶^median (IQR), *n* = 8 per group.

^*∗*^ *p* values were calculated using one-way ANOVA or the Kruskal-Wallis test. A *p* value < 0.05 indicates a statistically significant difference between the investigated groups.

^a^ *p* < 0.005 compared to group N; ^b^*p* < 0.05 compared to group PQ.
